# Free Energy Simulations of Receptor-Binding Domain Opening of the SARS-CoV-2 Spike Indicate a Barrierless Transition with Slow Conformational Motions

**DOI:** 10.1021/acs.jpcb.3c05236

**Published:** 2023-09-27

**Authors:** Victor Ovchinnikov, Martin Karplus

**Affiliations:** † Department of Chemistry and Chemical Biology, Harvard University, Cambridge, Massachusetts 02138, United States; ‡ Laboratoire de Chimie Biophysique, ISIS, Université de Strasbourg, 67000 Strasbourg, France

## Abstract

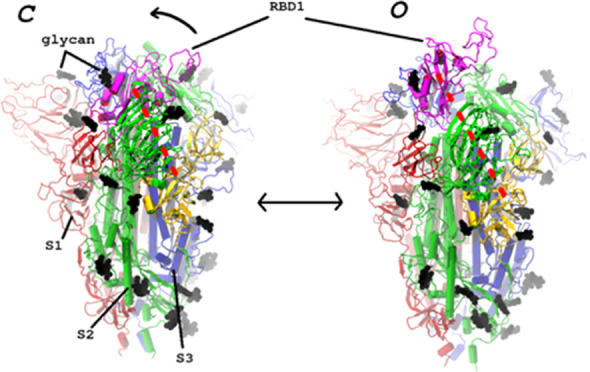

Infection by sarbecoviruses begins with the attachment of the homotrimeric viral “spike” protein to the angiotensin-converting enzyme 2 receptor on the surface of mammalian cells. This requires one or more receptor-binding domains (RBDs) to be in the open (up) position. Here, we present the results of long molecular dynamics simulations with umbrella sampling (US) to compute a one-dimensional free energy profile of RBD opening/closing and the associated transition times. After ≃3.58*μs* of simulation time per US window (∼229 μs in total), which was required to approach trajectory decorrelation, the computed free energy profile was found to be without large barriers. This suggests that the RBD diffuses between the open and closed positions without significant energetic hindrance. This interpretation appears consistent with experiments but is at odds with some previous simulations. Modeling the RBD motion as diffusive dynamics along the computed free energy profile, we find that the overall time required for the transition is only about 2 μs, which is 5 orders of magnitude shorter than experimentally measured transition times. We speculate that the most likely reason for the transition time mismatch is our use of very short glycans, which was required to make the simulations performed here feasible. Despite the long simulation times, the final free energy profile is not fully converged with statistical errors of ≃1.16 kcal/mol, which were found to be consistent with the slow time decay in the autocorrelation of the conformational motions of the protein. The simulation lengths that would be required to obtain fully converged results remain unknown, but the present calculations would benefit from at least an order-of-magnitude extension.

## Introduction

The COVID pandemic of 2019 resulted in the rapid development of highly effective vaccines against the SARS-CoV-2 coronavirus,^1^ with an effectiveness of ≥90% against the original (Wuhan) strain.^2,3^ However, mutations in the coronavirus spike protein,^4^ compounded by high transmission rates^5^ and selective evolutionary pressure exerted by vaccine-induced antibodies, continue to drive the emergence of viral “escape” variants, against which the antibodies induced by the standard prime-boost vaccination regimen had much reduced efficacy, e.g., 67–70% against the Omicron variant.^6^

As the descendants of the original Wuhan strain of COVID-19 are likely to become endemic,^7^ there is an unmet need to develop coronavirus vaccines capable of raising broad-based immunity to different strains, which would hopefully include some that are yet to emerge. To address this challenge in the framework of rational antigen design, a detailed molecular-level understanding of the determinants of coronavirus infectivity is necessary. Because infection with sarbecoviruses begins with the attachment of the trimeric viral spike to the angiotensin-converting enzyme 2 (ACE2) receptor found in many cells throughout the body, the spike is the target of most antibodies that neutralize COVID-19. The binding occurs via an exposed receptor-binding domain (RBD), which is relatively loosely attached to the rest of the spike (stem), as different positions and orientations of the RBD have been inferred from solution studies.^8−10^ The dynamics and energetics of RBD motion between the two positional extremes, “closed” (RBD down) and “open” (RDB up), appear to have a significant effect on the virulence of the virus. For example, the mutation D614G that is near the base of the attachment of the RBD to the spike increases the prevalence of open configurations,^10^ which are believed to be responsible for the higher infectivity of this strain.^11^ However, open positions also expose the RBD to the immune system, which elicits the maturation of anti-RBD antibodies, increasing the evolutionary pressure on the virus to mutate to avoid immune detection.

While the majority of the information needed for rational coronavirus vaccine design comes from experiments in structural biology and biochemistry, molecular dynamics (MD) simulations are increasingly able to aid in the interpretation of such experiments by providing dynamic information on temporal and spatial scales that are finer than the resolution of the experiments. In the context of rational vaccine design, it is useful to be able to predict computationally how amino acid mutations affect the distribution of antigenic epitopes that are exposed to the immune system. For example, stabilization of antigenic spikes in the prefusion conformation^12^ is often used to increase vaccine efficacy. Here, we present results from long MD simulations that use umbrella sampling to compute the free energy (FE) profile and the associated rates via diffusion theory along a 1D reaction coordinate of RBD opening in the proline-stabilized SARS2 spike mutant.^9^ The main purpose of the study is 2-fold: (i) to establish the amount of sampling required to obtain converged FE profiles in such a large and partially disordered biomolecular system and (ii) to explain the origin, at the atom level, of the rather high (≥5 kcal/mol) free energy barriers to opening that have been observed in some earlier studies. To further motivate this study and to place it into proper context, we summarize in the following the computational studies that are most relevant to our work. The informed reader may wish to skip to the next section.

## Previous Simulations

Sztain et al.^13^ investigated the closed-to-open (C↔O) transition in a fully glycosylated spike model^14^ using the weighted ensemble (WE) method. Briefly, multiple unbiased simulations were launched in parallel from the closed state and run for 100 ps; at the end of this iteration, the trajectory which had progressed the farthest toward the open state, on the basis of a preselected 2D progress coordinate, was used to reinitialize the next trajectory ensemble. The process was repeated until the RBD of the simulated spike sampled the open conformations. Several open conformations were observed that were similar to previously determined experimental cryo-EM structures.^15^ The authors also reported that typical transition lengths were hundreds of nanoseconds. However, because of the basing inherent in WE, they could not be related to the transition rates or relative populations of different states. Consequently, these results were not reported. An important prediction from the simulations was that a glycan at position N343 promotes RBD-open conformations. This appears to be consistent with an experimental measurement that showed reduced ACE2 binding by the N343A mutant.^13^

Gur et al.^16^ proposed a model of the C↔O transition on the basis of steered MD (SMD)^17^ simulations. Starting from intermediate structures obtained from SMD, the authors generated conformational ensembles using unbiased MD. They were projected onto principal components having the highest variance, which were computed from MD simulations of the end points, to visualize a 2D transition landscape. The authors did not describe whether they removed the bias arising from the use of the steering forces in generating the intermediate trajectories. However, they reported a metastable state, which may be consistent with experiments.^8^ Another approach using SMD simulations was reported by Ray et al.,^18^ but their nearly monotonic FE profiles suggest that the transition conformations sampled are far from equilibrium.

Fallon et al.^19^ computed an initial pathway for the C↔O transition making use of the equilibrated structures of Casalino et al.^14^ The pathway was represented by 32 backbone structures of the spike, computed using the nudged elastic band (NEB) method,^20^ followed by SMD in the full coordinate space to steer an all-atom structure to each of the NEB structures. The resulting pathway was input as initial conditions for 2D umbrella sampling (US) simulations, which were then used to construct 2D free energy landscapes spanning the C↔O transition for the wild-type (WT) spike as well as for three mutants. The NEB method for path finding has the advantage that it does not introduce directional bias, unlike steered or targeted dynamics,^21^ and potentially requires less MD equilibration to converge to paths of high equilibrium probability. With around 300 US windows, each simulated for 16 ns for each mutant, the authors could only interpret the FE landscapes qualitatively because of the high uncertainties in modeling and statistics. Generally, the simulations revealed landscapes with relatively shallow basins, the open and closed states being separated by barriers of between 2.5 and 5 kcal/mol and differing in free energy by −3 to 3 kcal/mol, depending on the mutant simulated.

Wu et al.^22^ used parallel cascade MD,^23^ a variant of the WE method, to generate a C↔O transition pathway, which was subsequently used to initialize US simulations. The reaction coordinate was the root-mean-square difference (RMSD) between the simulation and the open structure. Unfortunately, some important simulation details were not clearly stated, such as whether the simulation structures included glycosylation, the duration of sampling within the US windows, and the type of statistical or error analysis used. The FE profile computed for the transition showed that the closed structure is more stable by ≃3 kcal/mol than the open structure. However, the RMSD used as a reaction coordinate can be associated with a large Jacobian correction factor due to the coordinate transformations from Cartesian coordinates (see, e.g., ref (24)), which makes quantitative comparison to other studies difficult.

Pang et al.^25^ performed US simulations to characterize the C↔O transition for three SARS-CoV-2 spike variants, the fully glycosylated wild-type (WT), the deglycosylated WT spike, and the deglycosylated double proline mutant.^9,15^ For each system, a partial 2D landscape containing the minimum-free-energy path was computed. It was composed of 1000–1200 windows, with total simulation lengths of 12–91 μs, depending on the spike variant. To improve convergence of the US simulations, the authors used Monte Carlo (MC) replica exchange, in which adjacent windows can swap their bias values, according to the Metropolis MC criterion.^26^ With this method, the authors report impressively low statistical errors, most of which are in the range 0.1–0.15 kcal/mol. However, for the fully glycosylated spike, they predicted the open conformation to be unfavorable by 5.2 ± 0.1 kcal/mol, which appears to be at odds with the experimental observations of equal populations of the spike in the two positions, inferred from fluorescence resonance transfer (FRET) data^8^ and analysis of cryo-EM images.^9,10^ It is noteworthy that the glycan-free simulations correctly predict the two conformations to be nearly isoenergetic,^25^ but in view of the importance of glycans noted in other studies, this finding may not be biologically significant. The authors also computed the mean first passage time (MFPT) for the C↔O transition in the systems and noted that their high value of ∼1.5s in the forward direction, compared to ∼0.3s from the experiment of Lu et al.,^8^ could be caused by insufficient sampling of glycan conformations. However, the most likely cause of the long MFPT in the calculations of Pang et al.^25^ could be the high FE barrier, which is 11 ± 0.1 kcal/mol. By contrast, the distribution of fluorescence intensities that were used to identify the degree of spike opening was essentially flat,^8^ suggesting the absence of significant barriers. However, the “reaction coordinates”, i.e., the variables chosen to measure the extent of opening, were different between the calculation and the experiment, so that the two profiles may not be directly relatable.

The above listing of studies illustrates the challenge of reconciling computational with experimental studies and also of different computational studies with each other. Further, the existing simulations have not established the extent of sampling required to obtain converged FE profiles in this large and partly disordered biomolecular system; e.g., the 12*μs* simulations of Pang et al.^25^ appear similarly converged as the 91μs ones. They have also not explained the molecular origins of the (≥5 kcal/mol) free energy barriers to RBD opening or closing that have been observed. In this study, we provide more detail on the energetics and dynamics of the RBD transition using very long free energy simulations in one dimension.

## Methods

To prepare initial structures for the umbrella sampling (US) simulations, we used the zero-temperature string (ZTS) method,^27^ as implemented in CHARMM.^28,29^ Specifically, we refined by minimization of an all-atom linearly interpolated path between the C (PDB: 6VXX)^9^ and *O* (PDB: 6VSB)^15^ end points, with the missing loops modeled by Casalino et al.^14^ This initialization approach is chosen because it does not introduce directional bias into the path^21^ and is similar to the NEB method used by Fallon et al.^19^ The ZTS was represented by 64 discrete structures (replicas). To generate the interpolant while avoiding steric clashes, we used the method of Noé et al.,^30^ in which the side chains are shrunk before interpolation (here, to one-third of their original size), and the equilibrium bond lengths are restored automatically during subsequent minimization.

Previous simulations of the fully glycosylated spike protein immersed in a periodic box of solvent required more than 750 K atoms,^25^ which included only 63K protein and glycan atoms, and the remaining were solvent atoms. To reduce the calculation cost and memory demand, we performed the US simulations using a quasi-equilibrium solvation shell model,^31^ in which the spike was surrounded by a thin layer of solvent, whose evaporation was prevented using restraint potentials implemented as a plugin for the MD OpenMM library.^32^ The solvation model has been validated in unbiased MD, as well as free energy simulations, and found to introduce only minor artifacts, in the form of a slight but controllable departure from equilibrium dynamics, and increased solvent pressure on the biomolecule.^31^ Comparisons of potential of mean force (PMF) profiles of antigen–antibody separation produced with the shell model vs the periodic solvent box model showed that the differences between them were within statistical error^31^ (see also Figure S5 in the Supporting Information). Sodium and chloride ions were added to the solvent shell to achieve a total ion concentration of 150 mM.

As mentioned in the Introduction section, prior simulations of the fully glycosylated SARS-CoV-2 spike gave results that appear inconsistent with experiments, which was attributed to a lack of convergence of glycan motion sampling.^25^ To avoid this problem, we truncated all glycans to the N-acetylglucosamine moieties present in the PDB coordinates; however, the use of the short glycans also implies that our system better approximates the deglycosylated spike. In addition, we replaced the sequence containing the furin site (677–688) that was unresolved in the PDB (but modeled by Casalino et al.^14^) with the linker GSA at residue position 677. The use of the linker is unlikely to impact the properties of the spike; Gobeil et al.^33^ found that the removal of the furin site resulted in a spike that was similar to the wild-type in terms of thermal stability and binding to ACE2.

The use of shorter glycans, the linker, and the solvent shell layer of 10 Å resulted in relatively small solvated systems, ranging from ≃125 to ≃130 K atoms in total, depending on the ZTS replica (1–64) and permitting over two hundred microseconds of aggregate MD simulation time. The solvated systems were simulated using the OpenMM^32^ library with the CHARMM36 energy function^34,35^ in TIP3 water.^36^

The MD simulation setup included particle-mesh Ewald (PME) electrostatics, a 9 Å nonbonded cutoff for Lennard-Jones interactions, and hydrogen-mass repartitioning, which transfers 3 amu to every hydrogen from the parent heavy atom, reducing corresponding angle vibration frequencies and allowing a 4 fs simulation time step. Covalent bonds to hydrogens and water molecules were kept rigid, and the equations of motion were integrated using a Langevin dynamics integrator at 300 K with the atomic friction set equal to 1 ps^–1^ for plain US simulations and to 5 ps^–1^ for computing diffusion constants. The reason for the higher value used in computing the diffusion constants was to reduce the enhancement of self-diffusion by the TIP3 water model.^37^

**Figure 1:**
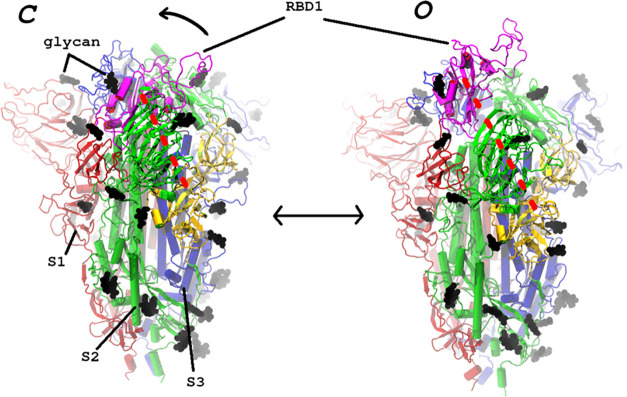
SARS-CoV-2 spike structures used in this study. Left: closed (all down) conformation;^9^ right: open (one-up) conformation;^15^ the coloring scheme is as follows: the spike protomers are labeled S1–S3 (i.e., S2 refers to protomer 2, *not* to the S2 subunit of a monomer downsequence of the furin cleavage site) and are drawn in red, green, and blue, respectively; the short glycans are drawn as black vdW spheres; the red dashed line indicates the reaction coordinate (RC), defined as the distance between S1 RBD (in magenta) and a part of S2 (in yellow). A black curved arrow indicates the direction of the RBD1 opening motion.

**Figure 2:**
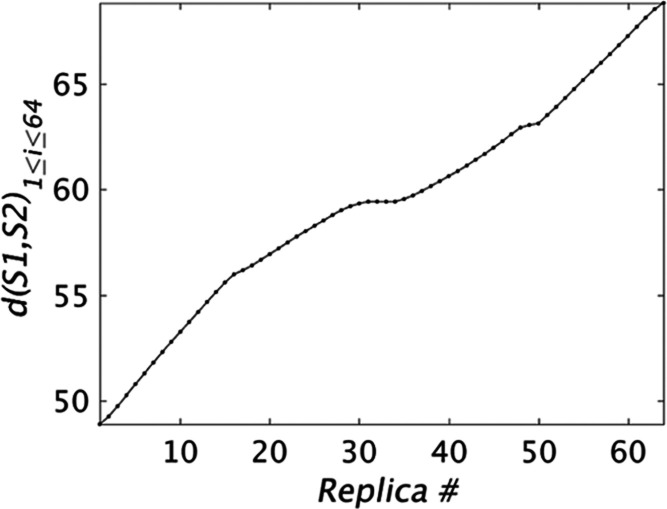
Evolution of the RC along the zero-temperature path for the *C*↔*O* transition. The RC is the distance between centers-of-mass of atom groups defined in the Supporting Information.

**Figure 3:**
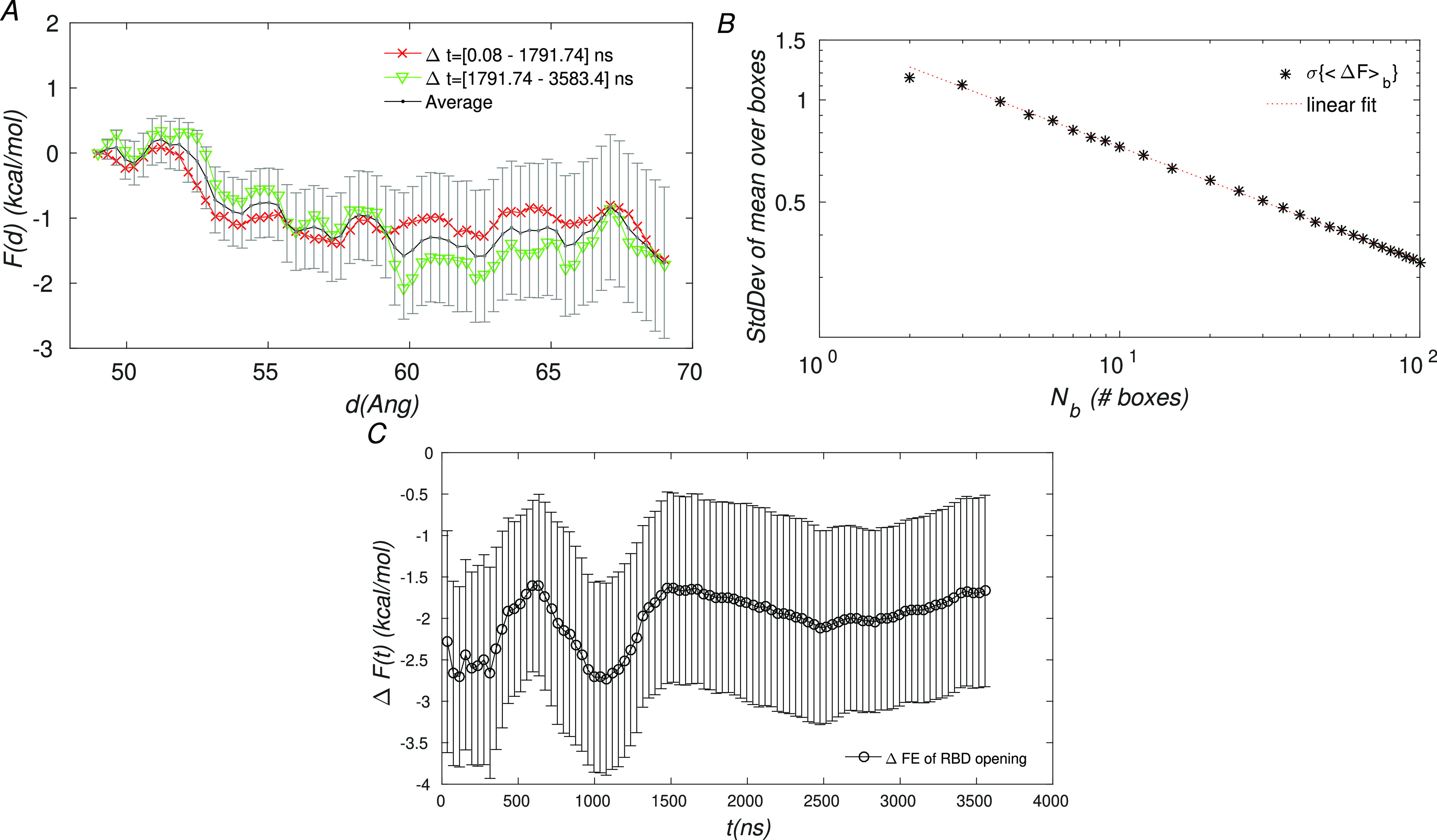
Free energy profile for the C↔O transition and its convergence behavior. (A) The final FE profile after ≃3.6μs of simulation per window; the gray error bars correspond to the standard error; for a more qualitative visualization of convergence, the statistics are also split into two consecutive blocks and FE profiles computed using data from each block. (B) Block average error analysis for correlated data^39^ indicates that the free energy time series have very long correlation times. (C) Time evolution of ΔFE_C→O_; the error bar is computed by splitting the sample at a time *t* into two parts, even though the samples are correlated, except possibly for the highest time value (≃3.6 μs).

The reaction coordinate (RC) for the opening motion was chosen to be the distance between the centers-of-mass (COMs) of the RBD of one monomer in the spike protein (RBD1), and a part of the spike formed by another monomer that excludes the RBD (STEM2ΔRBD2). This RC, which is similar to one of the coordinates used by Pang et al.,^25^ is illustrated in Figures 1 and 2 and is defined precisely in the Supporting Information. The chosen distance is convenient because it increases gradually from ∼49 to ∼69 Å during the transition, separating the end points and possible intermediates (see Figure 2). The free energy (FE) results shown in Figure 3 below also suggest the existence of several intermediate metastable states, which could correspond to those found by Lu et al.^8^

Umbrella sampling simulations used the harmonic flat-bottom restraints with a flat-bottom width of 0.5 Å and a force constant of 25 kcal/mol/Å^2^. The statistics from the flat-bottom (FB) part of the windows were used to compute the derivative of the FE profile under the assumption that it is linear over the FB window. Specifically,^38^ for a window with FB width Δ centered on *x*_0_, the FE derivative *F*′ is computed from the average of the RC value (*x̅*_MD_) in the FB portion of the window by numerically solving eq 1 for *F*′
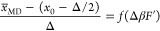
1where *f* is the monotonic function
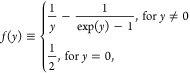
2β = (*k*_B_*T*)^−1^, where *k*_B_ is Boltzmann’s constant and *T* = 300 K is the temperature. The FE profile was obtained by integrating *F*′ using the trapezoid rule. Statistical uncertainty in *F*′ was estimated using the block average method for correlated time series.^39^

The first 4 nanoseconds of each simulation were performed with density control^31^ to maintain the solvent density in the outer solvation shell at ≃1g/mL by dynamically adjusting the solvent layer thickness.

Sixty-four equispaced US windows were used, with the C and *O* end points centered on *d* = 49 and 69 Å, respectively, which gives a window spacing of ≃0.3175 Å. Simulations corresponding to different windows were run in parallel, but asynchronously, on multiple computing nodes inside NERSC Perlmutter supercomputer, with each simulation using one NVIDIA A100 GPU and 16 AMD EPYC 7763 CPU cores, running at a speed of 90–115 ns/day, with the exact speed depending on the particular simulation window and overall system load. Using asynchronous, rather than synchronous parallel execution, as would be required with more sophisticated sampling strategies, e.g., replica exchange,^26^ allowed for up to an order-of-magnitude reduction in queue wait times.

Simulations were run in 20–25 ns increments while monitoring the FE profile for convergence using the blocking method for correlated time series.^39^ Simulations were stopped after 3583.4 ns (per window), at which point the block averages going from 3 to 2 blocks showed evidence of a nascent plateau, indicating a correlation length for FE derivatives of ∼1.5 μs.

To characterize the motion of the RC using the Smoluchowski diffusion model,^40^ we also computed the diffusion *D* coefficient in each RC window. Briefly, the simulation times at which the RC value crosses the boundaries of the flat-bottom (FB) regions are recorded, and the portions of the trajectories that are outside of the FB region are discarded and the simulation clock is stopped until the trajectory re-enters the FB region. In accordance with the diffusion model and the assumption of constant *F*′ over the FB region, the diffusion constant is related to the average roundtrip time between the FB boundaries^38^
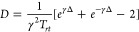
3where Δ is the FB width, γ = *F*′/(*k*_B_*T*), and *F*′ is the FE derivative in the simulation window. The above relation is accurate provided that the FE profile is well approximated by a line over the FB window. To investigate the effect of Δ, we performed 20–40 ns of MD simulations in each window with Δ = {0.5, 1, 1.5, 2}.

Principal component analysis (PCA) was performed essentially as in ref (41) but using coarse-grained residue coordinates, which are defined in the Supporting Information.

## Results

The free energy profile, block average analysis, and the running error estimate of the FE change of the C↔O transition are shown in Figure 3A–C, respectively. Figure 3A shows that the FE profile for the C↔O transition is without significant barriers, with ruggedness of the order ∼1 kcal/mol (1.7*k*_B_*T*). Although the statistical error in the profile remains rather high, reaching ∼1.16 kcal/mol at the O end point, it is sufficiently small that the O state is predicted to be lower in the FE than the *C* state (ΔFE_C→O_ = −1.69 ± 1.16 kcal/mol). This value is consistent with the result of Pang et al.,^25^ who show a profile with ΔFE_C→O_ ≃ −1.25 ± 0.2 kcal/mol. While experiments on the WT spike show a slightly favored C state (ΔFE_C→O_ ≃ 0.33 kcal/mol^8^), the K987P substitution in the proline mutant simulated here cannot form transient salt bridges with D427 and D428, both of which would stabilize the C state, as noted before.^25^ Furthermore, the FRET data of Lu et al.^8^ actually showed a higher energy state near the C state (which could be an alternative, more compact, conformation of the C state), for which the ΔFE_C→O_ is ≃ −0.27 kcal/mol. Thus, our computed ΔFE_C→O_, though not very precise, appears to be consistent with the existing data. However, the fact that we used short glycans may limit its biological significance.

It is noteworthy that the present FE profile and the FRET histograms of Lu et al.^8^ do not show significant transition barriers (i.e., ones significantly larger than the thermal energy *k*_B_*T* ≃ 0.6 kcal/mol). Qualitatively similar behavior was observed in simulations of the A522L/V mutants by Fallon et al.,^19^ whereas the more recent work of Pang et al.^25^ showed an ∼9 kcal/mol barrier, approximately 33% into the transition. (The corresponding barrier was 5.1 kcal/mol for the WT deglycosylated spike, which was simulated for ≃7.6 times longer than the proline mutant.) Although we also observed a small barrier of ≃1 kcal/mol, relative to the *C* state, in the first few hundred nanoseconds of simulation (Figure S1A–C), the barrier progressively disappeared as the duration of the simulation was increased.

Speculations about the reasons for the discrepancies in the FE barriers are difficult. One possible explanation is that different regions of conformational space are sampled by different methods. The differences begin with the choice of initial configurations for umbrella sampling. While the end point states are specified by PDB structures (excluding missing loops, which must be modeled computationally or taken from related PDB structures), intermediate structures are not known and thus must be created entirely by computation. Here, and in ref (19), the starting conditions are, roughly speaking, coordinate interpolants. By contrast, the methods used in refs (22) and (25) use dynamical simulation methods that bias the simulated structure toward^17,42,43^ or away from^44^ prescribed states using applied forces or selection. Our previous work on a much simpler protein suggests that the two types of methods can produce significantly different paths, with respect to the order of conformational events, as well as the energetics.^45^ Sampling differences could also arise from the use of different RCs. While we use a single distance, Pang et al.^25^ use an additional angle to describe the orientation of the RBD relative to the spike. However, their 2D FE landscapes suggest that integrating over the angle dimension would not substantially reduce the observed barrier. Fallon et al.^19^ also compute a 2D landscape, using a linear and a dihedral angle as coordinates; however, the barriers observed in their landscapes vary in the range 2–5 kcal/mol, depending on the mutation studied. Unfortunately, investigating the above possibilities in the current context would require systematically repeating simulations starting from different initial conditions, possibly with different restraints, which would be computationally prohibitive, as the present FE profile alone required ∼230 μs of simulation on a supercomputer.

The FE profile by itself is related only to the probabilities of observing a particular extent of RBD “openness”, as measured by the distance defined above. To obtain a kinetic description, we used the formalism of 1D diffusion in the overdamped regime^40^ to compute a position-dependent diffusion coefficient *D*, in addition to the FE profile.

**Figure 4:**
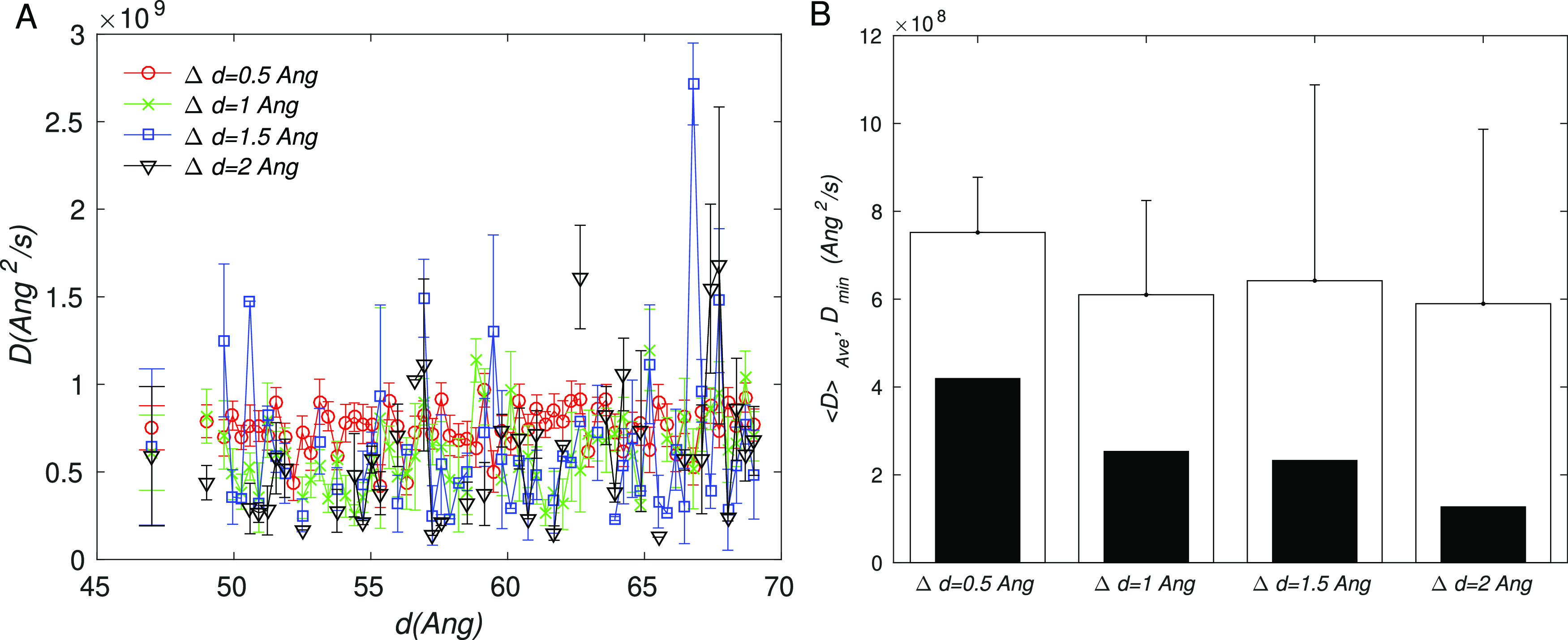
Diffusion constant computed from MD simulations using eq 3. (A) Diffusion constant along the RC (49–69 Å) for different values of the flat-bottom width Δ. The values plotted at *d* = 47 Å are averages over the RC range, which are also shown in part B as a bar graph. Error bars correspond to the standard error. (B) Transparent bars correspond to the average *D* over the RC; the error bar is the standard deviation; solid bars correspond to the minimum *D* over the RC.

The results of the diffusion calculations are summarized in Figure 4. It can be seen from Figure 4A that the computed *D* is variable across the RC, with the variability generally increasing with the size of flat-bottom window Δ. The source of the variability is mainly statistical uncertainty, which increases with Δ because longer times are needed to cross larger FB widths (the Taylor expansion of eq 3 leads to *T*_*rt*_ ≃ (Δ^2^ + *O*(Δ^4^))/*D*). Consequently, fewer crossing events are observed for larger Δ values in a fixed simulation time. For this reason, the simulations with Δ = 2 Å were twice as long as the others (40 ns per window). Further, there were windows for which we did not observe any crossing events; for those windows, we used linear interpolation to approximate the *D* from neighboring windows.

In spite of the high variations in *D* along the RC, the average diffusion constant ⟨*D*⟩ across all windows was similar for all values of Δ, varying in the range 6 × 10^8^ to 7.5 × 10^8^ Å^2^/*s* (Figure 4B). To provide a perspective for these values, we note that the self-diffusion constant of pure water at room temperature is 2.3 × 10^11^ Å^2^/*s*, and a theoretical estimate of *D* using Enstein–Stokes theory for rigid spheres is *D* = *k*_B_*T*/(6πη*r*) ≃ 10.8 × 10^9^ Å^2^/*s*, where η is the dynamic viscosity for water at 20 °C (≃1 mPa·s), and we took *r* to be the radius of gyration of the RBD, computed from PDB 6M0J(46) to be 18.64 Å. The fact that our value is an order of magnitude smaller than the Einstein estimate appears reasonable, given that the RBD is a flexible protein with residues and loops that can hinder motion by interacting with the rest of the spike.

**Figure 5:**
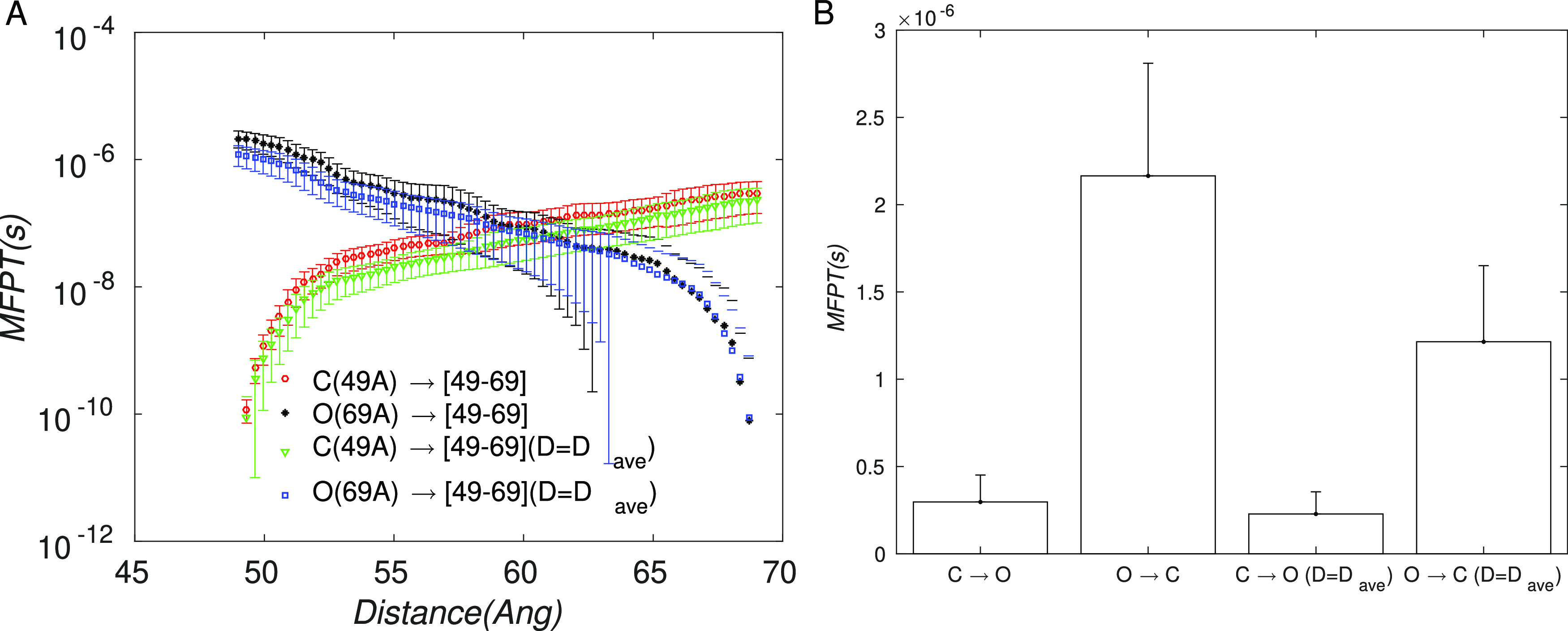
Mean first passage times (MFPTs) for the C↔O transition of RBD1. (A) In each direction, MFPTs computed from the end point to each intermediate point using the position-dependent diffusion coefficient and its average value are shown. (B) MFPTs computed from each end point to the other end point using position-dependent and average *D* are shown.

With the FE and *D* known, we calculate the mean first passage time (MFPT) from the end point states to any other state by evaluating the following equations^40^ using linear interpolation and the trapezoid rule integration
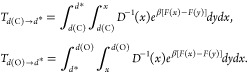
4In eq 4, *d*(C) and *d*(O) are the distances that correspond to the C and O states, respectively, and d* is any intermediate distance. The results of the integration are shown in Figure 5A, where we compare the C↔O and O↔C MFPT profiles computed using the instantaneous and the path-averaged diffusion coefficients; Figure 5B shows the MFPT values between the end point states.

First, from Figure 5, we note that using the average vs instantaneous *D* does not lead to significant differences between the MFPT profiles. This result is to be expected from eq 4, because the effect of *D* appears through its reciprocal, whereas the free energy *F* enters exponentially. Consequently, variations in *D* have a smaller effect than those in *F*. This observation also suggests that there is no compelling reason to improve the precision of the computed *D* profile using longer simulations before improving the precision of *F*.

Second, we note that the longest MFPT computed here is for the *O*↔*C* transition, corresponding to 2.2 ± 0.6 *μs* or the rate of 0.45 ± 0.12 μs^–1^ = 450 × 10^3^ s^–1^. The computed rate is more than 5 orders of magnitude larger than the experimental rate for the slowest substep in the transition,^8^ which was estimated at 1.56 ± 0.01 or 1.99 ± 0.02 s^–1^, depending on the type of nanoparticle used. A possible explanation for the large discrepancy is that *D* is significantly overestimated in the calculations because of the very short glycans used. Longer glycans could provide frictional resistance to RBD motion through many transient interactions with each other and with protein residues. Computing the diffusion constant for the RBD in a fully glycosylated spike using the present method would be prohibitively expensive. For example, the 40 ns long simulations used here to compute *D* for the deglycosylated spike would need to be orders of magnitude longer and would probably require substantially more solvent to maintain hydration of the glycans, regardless of their length or conformation. Such simulations might need to be performed on special computing hardware like the Anton2 computer.^47^

We note that the standard TIP3 water model is known to overestimate the rates of self-diffusion at the typical values used for Langevin friction in MD simulations with a Langevin thermostat.^37^ For example, the friction value of 1 ps^–1^ leads to an overestimate by a factor of ∼2 (*D*_self_ of ∼4.5 × 10^11^ Å^2^/s vs the experimental value of 2.3 × 10^11^ Å^2^/s). For this reason, as mentioned in the Methods section, in the diffusion calculations, we used the friction value 5 ps^–1^, which improves the calculated self-diffusion coefficient to ∼3 × 10^11^ Å^2^/s.^37^ Thus, we expected that the *D* computed for the RC motion would be sufficiently accurate that it could not explain the discrepancies in the MFPTs. To check this possibility further, we recomputed *D* along the RC using the Langevin friction value of 1 ps^–1^ (see Figure S6). We obtained the average *D* value of 1.09 × 10^9^ Å^2^/s, which is less than twice those obtained above with the Langevin friction of 5 ps^–1^ and indicates that the accelerated diffusion in the water model could not account for the MFPT underestimate.

Pang et al.^25^ computed the MFPTs for the C↔O transition in the deglycosylated spike equal to 143 and 497 μs in the forward and reverse directions, respectively. Using the FE profile from their publication and eq 4, we estimated a constant *D* value consistent with their results to be *D* ≃ 1.5 × 10^8^ Å^2^/s, which is roughly consistent with our values; it is similar to the smallest value of *D* ≃ 1.4 × 10^8^ Å^2^/s computed by us in the Δ = 2 Å case (see Figure 4B). Thus, the main reason for the longer MTPFs in their case is the presence of an FE barrier; it is not the difference in the diffusion constant. For completeness, we also computed the MFPT profiles using the smallest *D* ≃ 1.4 × 10^8^ Å^2^/s computed from our simulations (see Figure S2), which results in a modest increase of the O↔C MFPT to 5.6 ± 1.2 μs.

**Figure 6:**
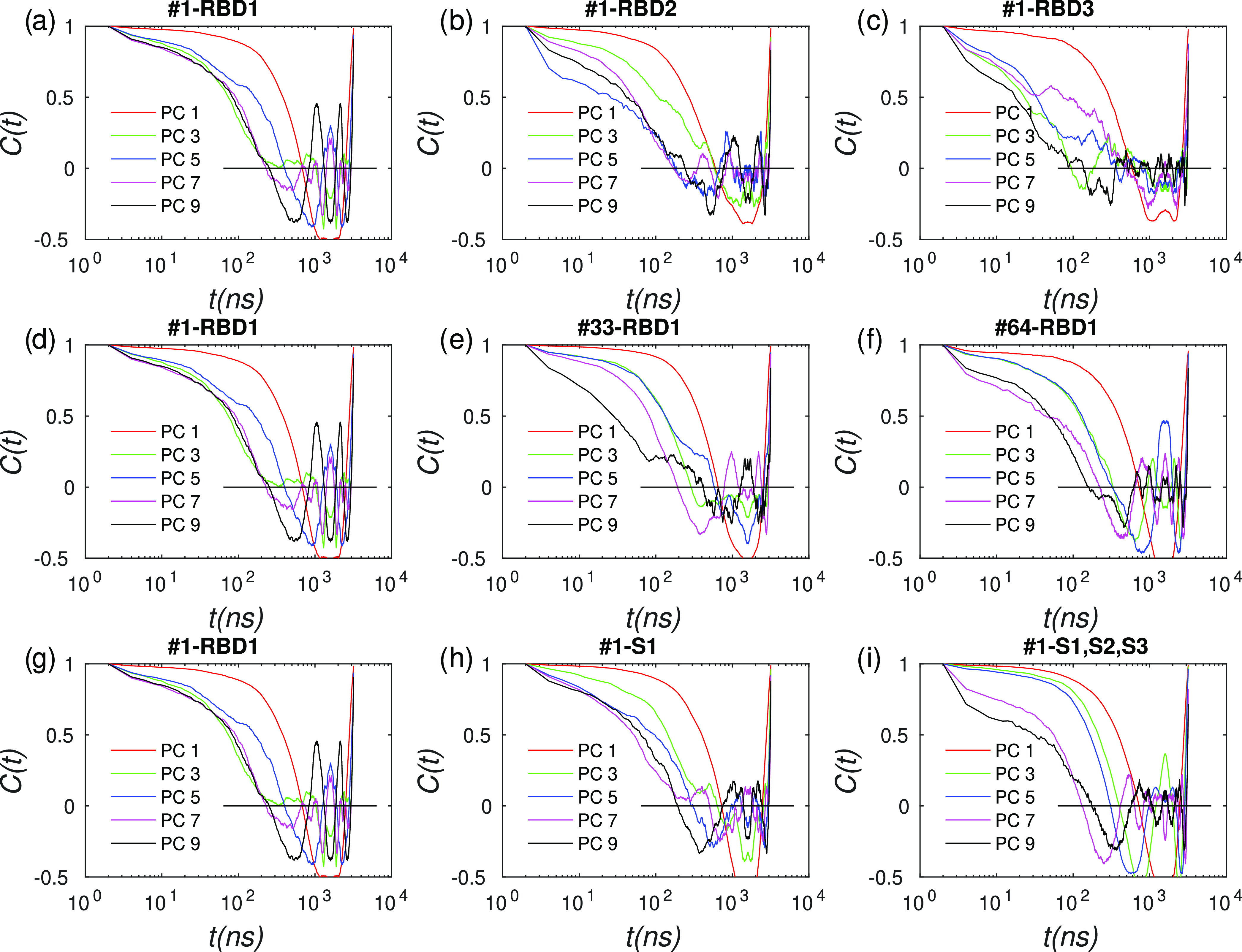
Autocorrelation functions (ACFs) of trajectory displacements along principal components (PCs) for various umbrella sampling (US) windows and spike subdomains. The superscript above each panel shows the US window number (1–64) followed by the subdomain tag; RBD1–3 correspond to the three receptor-binding domains, S1 corresponds to protomer 1 of the spike, and S1–3 correspond to the entire spike. PCs for each subdomain were computed after best-fit trajectory alignment using only the subdomain atoms, matrix diagonalization, and removal of rigid-body (0-frequency) modes. For clarity, only the ACFs corresponding to the first several low-frequency PCs are shown, specifically, modes #1,3,5,7,9.

To the best of our knowledge, the simulations performed here to compute the *C*↔*O* FE profiles are the longest reported for this transition. Specifically, each of the 64 windows was simulated for 3.583 μs, for a total of 229 μs. By comparison, Fallon et al.^19^ performed 16 ns of simulation in each of 328 or fewer windows (depending on the mutant) inside a 2D reaction coordinate landscape for a total of 5.25 μs. Pang et al.^25^ performed a total of 91 μs of simulation over 1211 windows in a 2D reaction coordinate landscape. We note, however, that they used Hamiltonian replica exchange^26^ to accelerate sampling by swapping RC values between adjacent windows, which could significantly improve conformational sampling. In spite of our long simulation times, we were surprised that the statistical error in the Δ*F* estimate remained rather high at 1.16 kcal/mol (see Figure 3). Further, Figure 3B suggests that the number of independent samples is only about 2, which corresponds to a trajectory decorrelation time of ≃1.8 *μs* and suggests the presence of long conformational memory. To relate this result to the time scales of conformational motions in the spike, we performed a principal component analysis (PCA) of the mass-weighted spike coordinates^41^ in each simulation window. To accelerate the matrix diagonalization required to compute the PCs, each residue was coarse-grained to a single particle located at the residue COM with a mass equal to the residue mass. Time scales of conformational motions can be visualized from the autocorrelation functions (ACFs) of displacements along PCs, as shown in Figure 6, for several PCs and spike subdomains.

The ACF for PC#1, which corresponds to the lowest-frequency motion in the quasiharmonic interpretation,^28^ crosses the vertical axis around 700–800 ns but does not decay to zero, instead showing anticorrelation. Higher PCs (#3–#9) show more rapidly decaying ACFs, with most of them fluctuating around zero in the neighborhood of 1 μs. Qualitatively, the results appear to be insensitive to the choice of domain considered. For example, the ACFs computed along PCs using a single RBD only (Figure 6a–c) show similar time scales as a spike monomer (Figure 6h) or the full trimer (Figure 6i), suggesting that the RBD alone exhibits slow conformational motions. This is not too surprising, as the RBD contains several loops that are partly disordered in solution,^9^ which needed to be modeled to perform MD simulations.^14^

To check that the long decay times in the ACFs were not introduced by the shell solvation model used here, we reanalyzed microsecond-long trajectories of the smaller proteins dihydrofolate reductase (DHFR) and myosin 6 (M6) studied before.^31^ Specifically, we compared the ACFs of PC displacements computed from simulations of these proteins in a periodic box filled with an explicit TIP3 solvent to those computed using the shell solvation model. The results are shown in Figures S3 and S4 for DHFR and M6, respectively, and indicate that the shell solvation does not increase correlation decay times. This conclusion is also consistent with the comparison of PMFs of antibody–antigen separation performed using a full box and a solvent shell, which gave similar results.^31^ To check how the PMF simulation length compared to the decorrelation length, we extended the simulations in ref (31) to ≃800 ns per window and applied block average analysis,^39^ which showed similar convergence behavior (Figure S5) for the two solvation treatments and statistically the same value for the PMF of separation.

A noteworthy feature of the very slow convergence behavior of Δ*F* in Figure 3C is that, after ≃1.5μs of simulation, it shows little variation, the value being confined to the range [−2.2, 1.6], with the standard deviation of the mean over two blocks around 1.2 (units of kcal/mol). Thus, it appears as though the last ≃2 microseconds of simulation do not actually improve the FE estimate. Instead, the additional simulation time serves to decorrelate the simulation samples, which requires very long integration times, possibly longer than those performed here. One positive feature of the apparent insensitivity of the FE profile to simulation duration is that an approximate estimate can be obtained within only a few hundred ns per window, albeit without a reliable statistical error, as the resulting trajectory is too short to contain multiple independent samples. The similar convergence behavior of the antibody–antigen separation FEs (Figure S5A,B) suggests that this feature may be common in conformational free energy simulations of proteins.

## Conclusions

The SARS-2 coronavirus infects cells after the receptor-binding domain (RBD) of its viral spike protein transitions to an “open” (or “up”) conformation, which is able to bind the angiotensin-converting enzyme 2 receptor, initiating viral cell entry. We have described the results of very long molecular dynamics simulations that use umbrella sampling (US) to compute the free energy (FE) profile and rates of transition between the two conformations along a 1D reaction coordinate (RC), chosen to be the distance between one of the RBDs and part of the stem of another spike protomer. The MD calculations were performed for ≃3.58 μs per US window, totaling 229 μs over 64 windows spanning the RC. The FE computed profile is relatively flat, with energy barriers of the order of 1 kcal/mol, which is broadly consistent with the experiment of Lu et al.^8^ and some of the calculations of Fallon et al.^19^ In contrast, Pang et al.^25^ found barriers of ∼5 and ∼8.5 kcal/mol, respectively, for the wild-type spike and the double proline mutant (the latter was simulated here). Despite the long total simulation times, which, to our knowledge, are the longest yet reported for this transition, the FE profile shows rather poor convergence, with statistical uncertainty for the FE difference equal to ≃1.16 kcal/mol. The large uncertainty is attributed to long trajectory decorrelation times, which suggests that strong memory effects are present in the conformational dynamics of the system. Consistent with this interpretation are the slowly decaying autocorrelation functions of displacements along the principal components (PCs) of the motions, which take hundreds of nanoseconds or longer to cross the horizontal axis and often remain negative for hundreds of nanoseconds longer. Modeling the transition as an overdamped diffusion on the FE profile, we computed the average diffusion constant associated with the motion along the RC to be around 6 ± 2 μm^2^/s, which is about four times higher than the value inferred from the simulation data of Pang et al.^25^ However, combined with the FE profile, this value yields predictions for the transition rate of about 450 × 10^3^ s^–1^, which is over 5 orders of magnitude faster than the experimentally measured result of ≃2 s^–1^(see ref (8)). We suggest that the main reason for the discrepancy is our use of very short glycans, which increases the simulation speed by reducing the total number of atoms. However, the simulations described here suggest that achieving configurational sampling sufficient for convergence of free energies of realistically large glycans^13,14^ may not yet be computationally tractable.

Slow decay of ACFs in protein simulations is not unexpected and has been observed before in MD simulations of smaller proteins, ranging in size from 21 to 101 kDa.^48^ For example, for the dimeric 416-residue protein phosphoglycerate kinase (PGK), ACFs of selected inter-residue distances decreased below 0.01 only after several microseconds; the authors interpreted the protein dynamics to be subdiffusive on a fractal energy landscape, which did not appear to be converged even after microseconds of MD simulations. Whether the simulations performed here could yield a converged and precise free energy profile using currently available computer resources remains unclear. However, at least an order-of-magnitude extension of the simulations presented here would almost certainly be required. In addition, the use of enhanced sampling methods for molecular simulation, especially those based on Hamiltonian replica exchange (HREX), could accelerate the decay in the ACFs, facilitating more rapid convergence. However, the exact degree of acceleration that would be achieved with HREX is not clear, as it is dependent on the simulation system, as well as on the simulation parameters, e.g., the number of replicas simulated concurrently, or the frequency of exchanges.

## Supporting Information Available

Data and analysis code for this study can be found online at https://zenodo.org/record/8200601. The Supporting Information is available free of charge at https://pubs.acs.org/doi/10.1021/acs.jpcb.3c05236.

Animation S1 accompany this article, which can be accessed online (MP4)
Supporting Methods, Figures S1–S6 (PDF)
