# Supplementary material for: Free Energy Simulations of Receptor-Binding Domain Opening of the SARS-CoV-2 Spike Indicate a Barrierless Transition with Slow Conformational Motions
Source: J Phys Chem B. 2023 Sep 27;127(40):8565–75. doi: 10.1021/acs.jpcb.3c05236 (PMC10578350; doi:10.1021/acs.jpcb.3c05236)
Supplement: Supplementary file 2 — jp3c05236_si_002.pdf [file jp3c05236_si_002.pdf]

Free Energy Simulations of Receptor-Binding Domain Opening in the SARS-CoV-2 Spike Indicate a Barrierless Transition With Slow Conformational Motions.

V. Ovchinnikov,[1,a] and M. Karplus[1,2,b]

[1] Department of Chemistry and Chemical Biology, Harvard University, Cambridge, MA, 02138, USA

[2] Laboratoire de Chimie Biophysique, ISIS, Université de Strasbourg, 67000 Strasbourg, France

[a] ovchinnv@georgetown.edu

[b] marci@tammy.harvard.edu

Supporting Figures

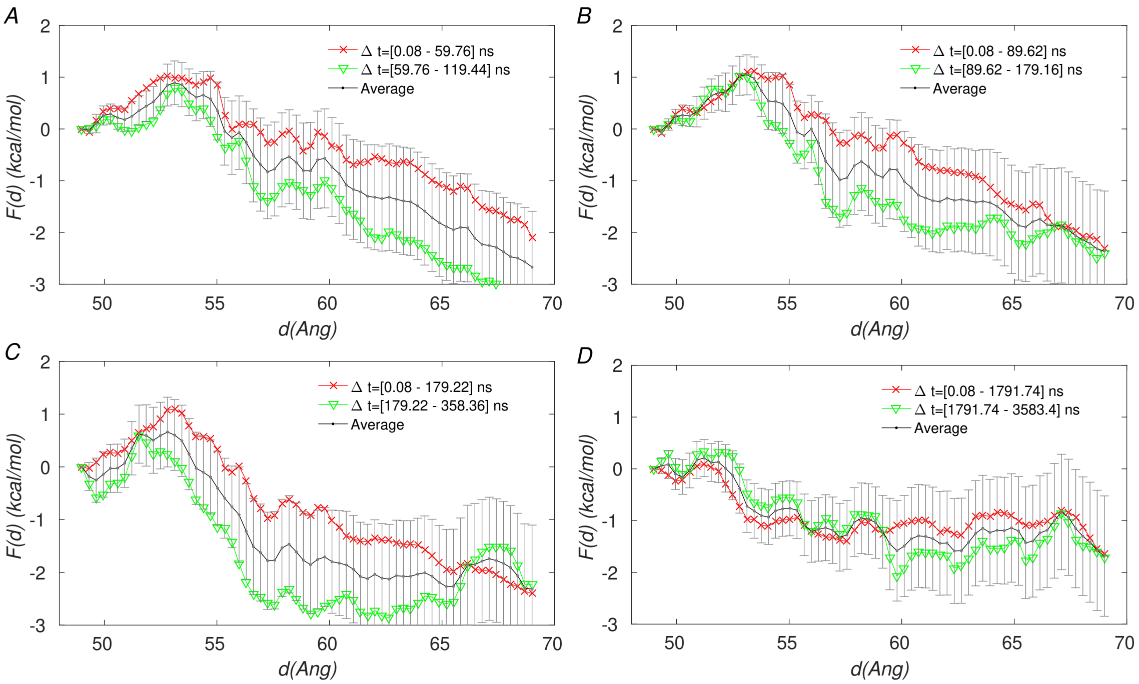

Figure S1: Free energy profile for the $C \leftrightarrow O$ transition computed at different total simulation time points. A: $t \leq 119.44ns$; B: $t \leq 179.16ns$; C: $t \leq 358.36ns$; D: $t \leq 3583.4ns$; as for Fig. 3A, the statistics are also split into two consecutive blocks, and FE profiles computed using data from each block; gray bars correspond to the standard deviation of the box averages over the two blocks.

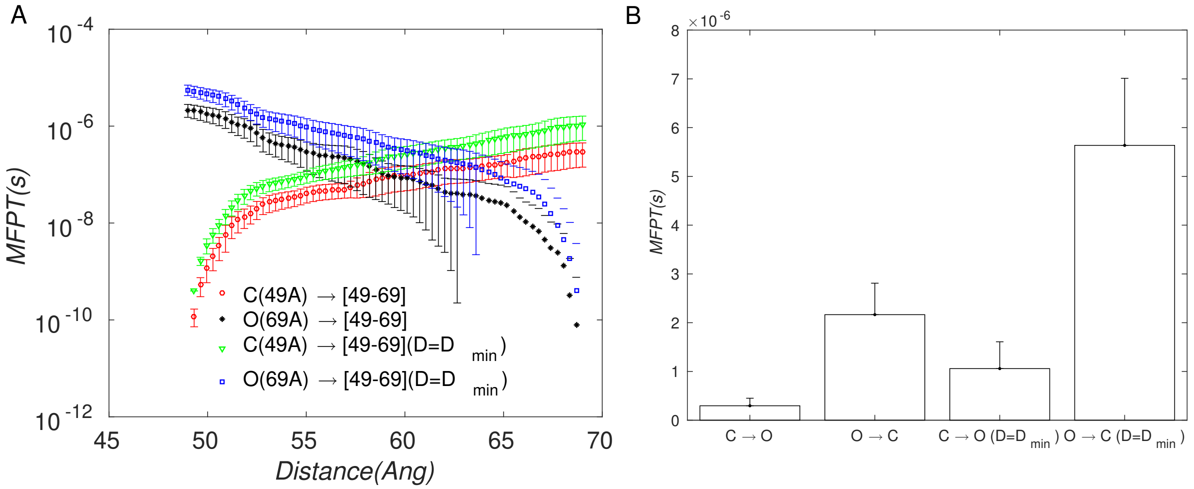

Figure S2: Mean first passage times (MFPTs) for the $C \leftrightarrow O$ transition of RBD1. A: In each direction, MFPTs computed from the end point to each intermediate point using the position-dependent diffusion coefficient, and the minimum value over the RC range, are shown. B: MFPTs computed from each endpoint to the other end point using position-dependent and minimum D are shown.

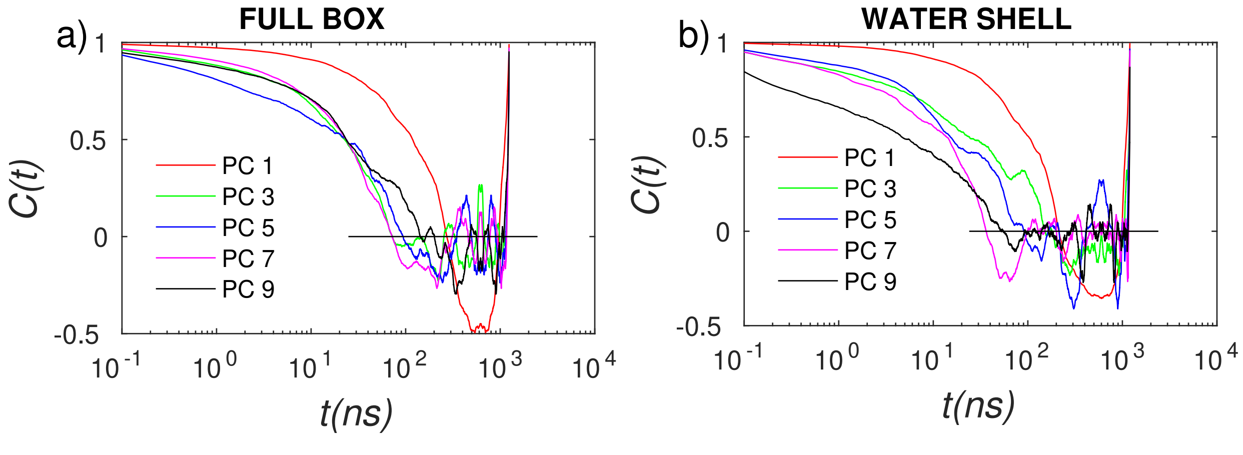

Figure S3: Comparison of autocorrelation functions (ACFs) of trajectory displacements along principal components (PCs) computed from MD simulations of dihydrofolate reductase performed in Ref. 31; a) simulation in a periodic box full of solvent ; b) simulation in a water shell. For clarity, only the ACFs corresponding to the first several low-frequency PCs are shown, specifically, modes #1,3,5,7,9.

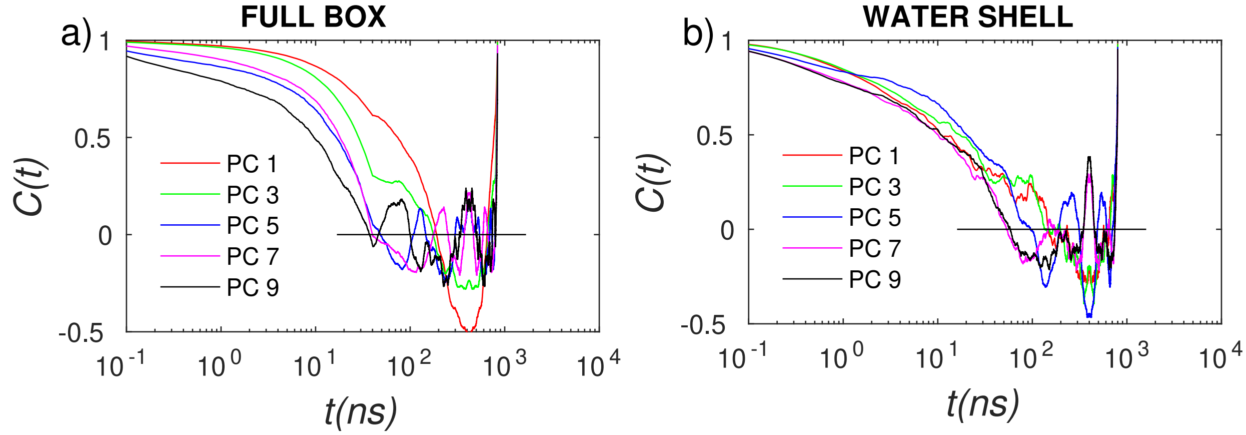

Figure S4: Comparison of autocorrelation functions (ACFs) of trajectory displacements along principal components (PCs) computed from MD simulations of myosin VI performed in Ref. 31; a) simulation in a periodic box full of solvent; b) simulation in a water shell. For clarity, only the ACFs corresponding to the first several low-frequency PCs are shown, specifically, modes #1,3,5,7,9.

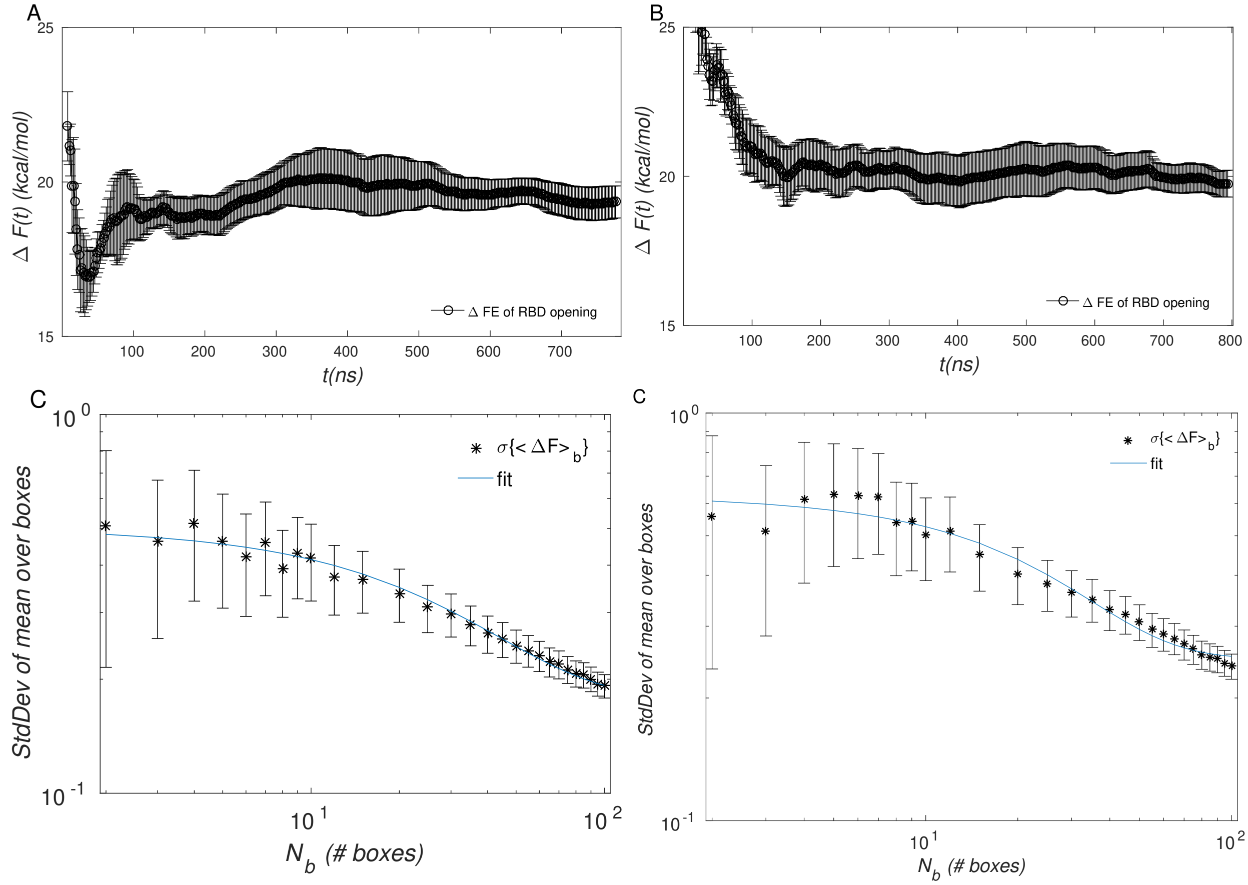

Figure S5: Comparison of the free energy of separation of an H1 influenza hemagglutinin antigen and antibody CR9114 ($\Delta F_{AG/AB}$) computed from MD simulations performed in Ref. 31; A,C: simulation in a periodic box full of solvent (ΔF=19.37 ± 0.47); B,D simulation in a water shell (ΔF=19.76 ± 0.58); A,B: Time evolution of $\Delta F_{AG/AB}$; the error bar is computed by splitting the sample at a time t into two parts, even though the samples are correlated for large values of N_b, as shown in panels C & D below; C,D: Block average error analysis for correlated data.[39]

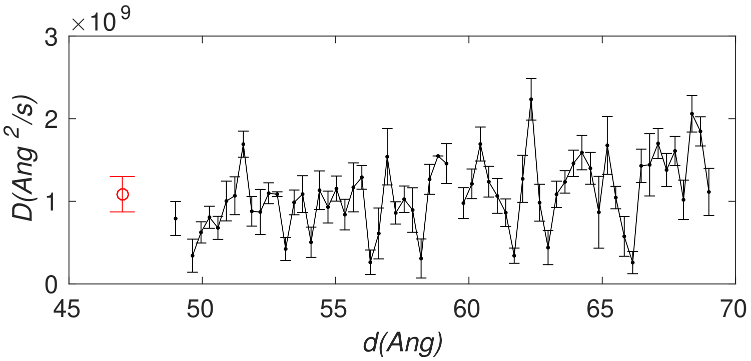

Figure S6: Diffusion constant computed from MD simulations performed with Langevin friction of 1ps^{-1} using Eq. (3). The value at d=47Å is the average over the RC range. Error bars correspond to the standard error.

Supporting Methods

Reaction coordinate definition

The reaction coordinate (RC) for the umbrella sampling simulations performed here was defined as the distance between the centers-of-mass (COMs) of the $C\alpha$ atoms of two domains. The first domain was the receptor binding domain of the first spike protomer, according to the definition in the PDB file 6VXX,[9] specifically, the $C\alpha$ atoms of residues 332–526. The second domain was a part of the viral stem of the second spike protomer, specifically, the $C\alpha$ atoms of residues 300–331 and residues 527–700. The subdomains are illustrated in Fig. 1 of the main text. The use of only the $C\alpha$ atoms increases the speed of applying the restraints in the MD calculation, because forces need to be applied to many fewer atoms (on average, there are about 19 atoms per amino acid); it also makes the COM equivalent to the center of geometry.

Autocorrelation of motions along principal components

To estimate the time scales of the slowest conformational motions in each umbrella sampling (US) window, we first computed principal components (PCs) of protein motion in coarse grained (CG) coordinates, as follows.

For each amino acid residue indexed by i composed of n_i atoms, we computed the CG coordinate triplet r_i^{CG} as

$$r_i^{CG} = \frac{\sum_{j=1}^{n_i} r_i^j m^j}{m_i^{CG}}, \tag{S1}$$

where

$$m_i^{CG} = \sum_{j=1}^{n_i} m_i^j, \tag{S2}$$

and r_i^j and m_i^j are the coordinate triplet and mass of atom j of residue i, respectively.

The CG coordinates were computed for each trajectory in each US window, with trajectory frames sampled in 100ps time increments. Next, the CG coordinates were shifted

to their COMs, and rotated to achieve the best-fit superposition,[49] *i.e.*, for each trajectory frame k

$$ {}^{k}r_i^{CG} \mapsto {}^{k}r_i^{CG} - \frac{\sum_{i=1}^{N_{res}} {}^{k}r_j^{CG} m_j^{CG}}{\sum_{i=1}^{N_{res}} m_j^{CG}}, \tag{S3} $$

followed by

$$ {}^{k}r_i^{CG} \mapsto A\ {}^{k}r_i^{CG}, \tag{S4} $$

where A is the best-fit rotation matrix defined as the minimizer

$$ A = \arg\min_{B \in M_{3x3}} \|B\ {}^{k}r_i^{CG} - \langle\ {}^{l}r_i^{CG}\rangle_{1\le l\le N_{fr}}\|, \tag{S5} $$

and angle brackets represent averages over the N_{fr} trajectory frames. Equation (S5) is applied iteratively five times to achieve self-consistency.

The mass-weighted covariance matrix of CG coordinate displacements is computed as

$$ C_{ij} = (m_i^{CG} m_j^{CG})^{1/2} \langle (r_i^{CG} - \langle r_i^{CG}\rangle)(r_j^{CG} - \langle r_j^{CG}\rangle)\rangle, \tag{S6} $$

where the angle brackets represent trajectory averages, as above.

The matrix C_{ij} is diagonalized in Matlab[50] using singular value decomposition routines to yield the diagonal eigenvalue matrix Λ (here, equivalent to the singular values) and eigenvectors (mass-weighted principal components) U,

$$ C = U \Lambda U^{-1}. \tag{S7} $$

Six of the singular values were very close to zero, and the associated modes, which correspond to rigid-body motion, were discarded. The mass-weighted coordinate projection onto PC $\#i$

is computed as

$$\eta_i = \sum_{j=1}^{N_{res}} U_{ij}(r_j^{CG} - \langle r_j^{CG} \rangle)\sqrt{m_j^{CG}}. \tag{S8}$$

We note that in the above Eqs. (S6) and (S8) to simplify notation we tacitly ignored the fact that each r_i is a coordinate triplet. To be precise, the residue indices should be interpreted as also running sequentially over the three Cartesian components (with the undesrtanding that the mass assigned to each component is the same). The autocorrelation functions of η_i were computed using the fast Fourier transform routine in Matlab.[50]
